# Influence of Non-invasive Ventilation in an Adult Patient With Acute Epiglottitis: A Case Report

**DOI:** 10.7759/cureus.30595

**Published:** 2022-10-22

**Authors:** Wail Bamadhaf, Ali AlRahma, Khalid Ali, Khaled Hamoud

**Affiliations:** 1 Emergency Department, Rashid Hospital, Dubai, ARE

**Keywords:** non-invasive ventilation, type ιι respiratory failure, adult epiglottitis, case report, non-invasive mechanical ventilation

## Abstract

Adult acute epiglottitis is a rare, life-threatening disease that requires prompt airway management, resuscitation, and stabilization. This case report discusses a patient with type ΙΙ respiratory failure due to acute epiglottitis, which was managed successfully with non-invasive positive-pressure ventilation in the emergency department.

We report a case of a male patient in his 30s who arrived at the emergency department complaining of sore throat, cough, and shortness of breath. He was found to be in severe respiratory distress. The patient’s condition improved dramatically after non-invasive positive-pressure ventilation, which prevented the need for endotracheal intubation. He was admitted to the ICU and discharged without complications five days later.

Non-invasive positive-pressure ventilation may help manage moderate cases of acute epiglottitis with partial airway obstruction as a bridging therapy until other treatments have decreased edema, thus preventing the need for endotracheal intubation.

## Introduction

Adult epiglottitis is a life-threatening disease with an overall incidence of 2.54 per 100,000 patients [1]. A growing body of literature indicates that epiglottitis is increasing among adults in the post-Hib vaccine era [2]. Epiglottitis or, more correctly, supra-glottitis, is a cellulitis of the structures above the glottis: the epiglottis, the aryepiglottic folds, the arytenoids, and the uvula [3].

Adult epiglottitis differs from pediatric epiglottitis in microbiology, presentation, and clinical course. While adult epiglottitis is generally considered more benign than epiglottitis in children, it remains a cause of acute airway compromise with a mortality rate ranging between 1% and 20% [2]. Maintaining the patient’s airway is the primary treatment for acute epiglottitis, along with early antibiotic therapy for infective etiology.

This article was previously posted to the Research Square preprint server on July 21, 2022.

## Case presentation

A male patient in his 30s arrived at our emergency department via private car for sore throat, cough, and shortness of breath. In the triaging area, the patient was in severe respiratory distress, with an oxygen saturation of 82% on room air. He was immediately shifted to the resuscitation area and connected to a non-rebreather mask. On examination, the patient seemed anxious and was sitting on the bed in a tripod position; peri-oral cyanosis was evident. He was found to have audible stridor and a muffled voice. He was only able to speak in one-word sentences, along with the use of accessory muscles during inspiration. His vital signs were heart rate: 118 BPM; blood pressure: 145/91 mmHg; respiratory rate: 31 bpm; and temp: 37.1 ˚C; his SpO2 improved to 89% with a 15 L non-rebreather mask. His throat was congested, but no soft tissue swelling or foreign body could be identified. Auscultation of the chest indicated fair air entry bilaterally with no added sound. Arterial blood gas was done (Table 1), and Anesthesiology was immediately notified of the possibility of difficult airway management.

**Table 1:** Arterial blood gas of the patient PH: Acid-base balance, PCO_2_: Partial pressure of carbon dioxide, PO_2_: Partial pressure of oxygen, HCO_3_: Bicarbonate, CTHB: Total hemoglobin, SO_2_: Oxygen saturation, FO_2_HB: Fraction of oxyhemoglobin, FCOHB: Fraction of carboxyhemoglobin, FMETHB: Fraction of methemoglobin, mmHg: Millimeters of mercury, mmol/L: Millimoles per liter, g/dL: Grams per deciliter, mg/dL: Milligrams per deciliter

Component	Result	Reference Range and Unit
PH	7.163	7.35-7.45
PCO_^2^_	55.1	35-45 mmHg
PO_2_	53.0	83-108 mmHg
HCO_3_	16.2	21-28 mmol/L
CTHB	15.1	13.8 -18.0 g/dL
SO_2_	77.9	95-99 %
FO_2_HB	76.3	95-98 %
FCOHB	1.2	0.5-1.5 %
FMETHB	0.9	0.5-1.5 %
Potassium	4.2	3.4-5.0 mmol/L
Sodium	144	134-143 mmol/L
Ionized Calcium	1.21	1.15-1.29
Chloride	113	97-108
Glucose	195	60-100 mg/dL
Lactic Acid	6.9	0.5-1.6 mmol/L

Due to the patient’s inability to speak, a collateral history was taken from family members. According to them, the patient developed a sore throat and cough 12 hours before arrival. Six hours later, he experienced sudden shortness of breath while having dinner.

**Figure 1:**
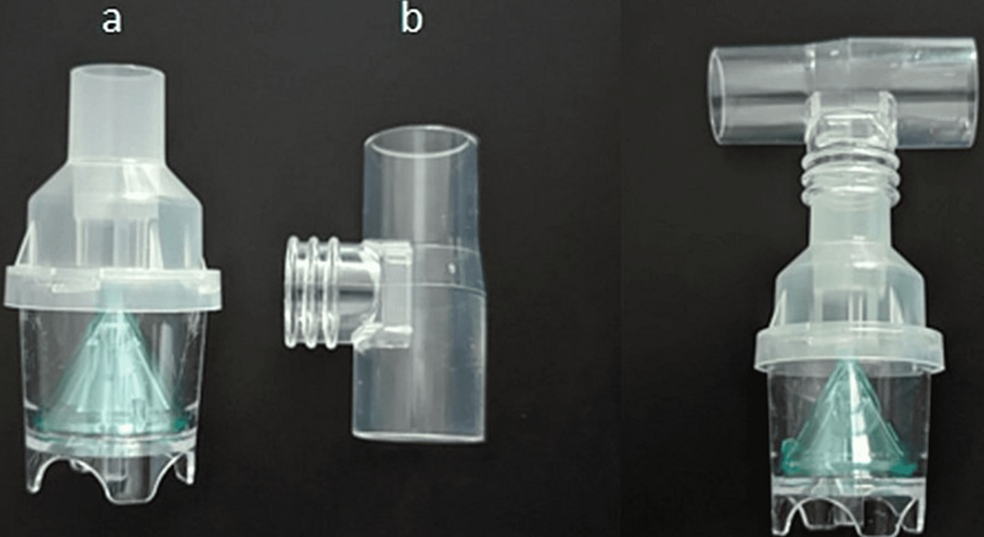
A t-piece instrument with nebulizer medicine chamber (a) Nebulizer medicine chamber, (b) T-piece instrument

After history and physical examination, our provisional differential diagnoses included epiglottitis, anaphylaxis/angioedema, and foreign body causing partial upper airway obstruction. Regarding anaphylaxis, the patient immediately received IM epinephrine, followed by chlorphenamine and dexamethasone. Ceftriaxone 1 g IV was given to treat the epiglottitis, and nebulized epinephrine was started. The patient showed minimal improvement with the above interventions, so it was decided to intubate him. However, to prevent cardiopulmonary collapse due to hypoxemia and the anticipation of difficult intubation, the patient was connected to non-invasive positive-pressure ventilation (NIPPV) via a face mask with a bilevel positive airway pressure (BiPAP) mode as a bridging step to increase his oxygen saturation and improve ventilation. The use of non-invasive ventilation led to a decrease in the patient’s work of breathing and an improvement in his anxiety level. It raised the oxygen saturation to 98% and dropped his RR to 22 bpm. This dramatic improvement led the attending staff agreeing to withhold intubation at that time. However, the team members were on standby with a double step-up intubation plan in case of any deterioration. The nebulized epinephrine continued while the patient was connected to the NIPPV via a T-piece instrument (Figures 1a, 1b).

A lateral mobile neck x-ray was ordered due to the patient’s critical condition looking for the “Thumb sign” to guide the diagnosis of Epiglottitis. Unfortunately, the x-ray was of poor quality due to the improper position of the patient.

**Figure 2:**
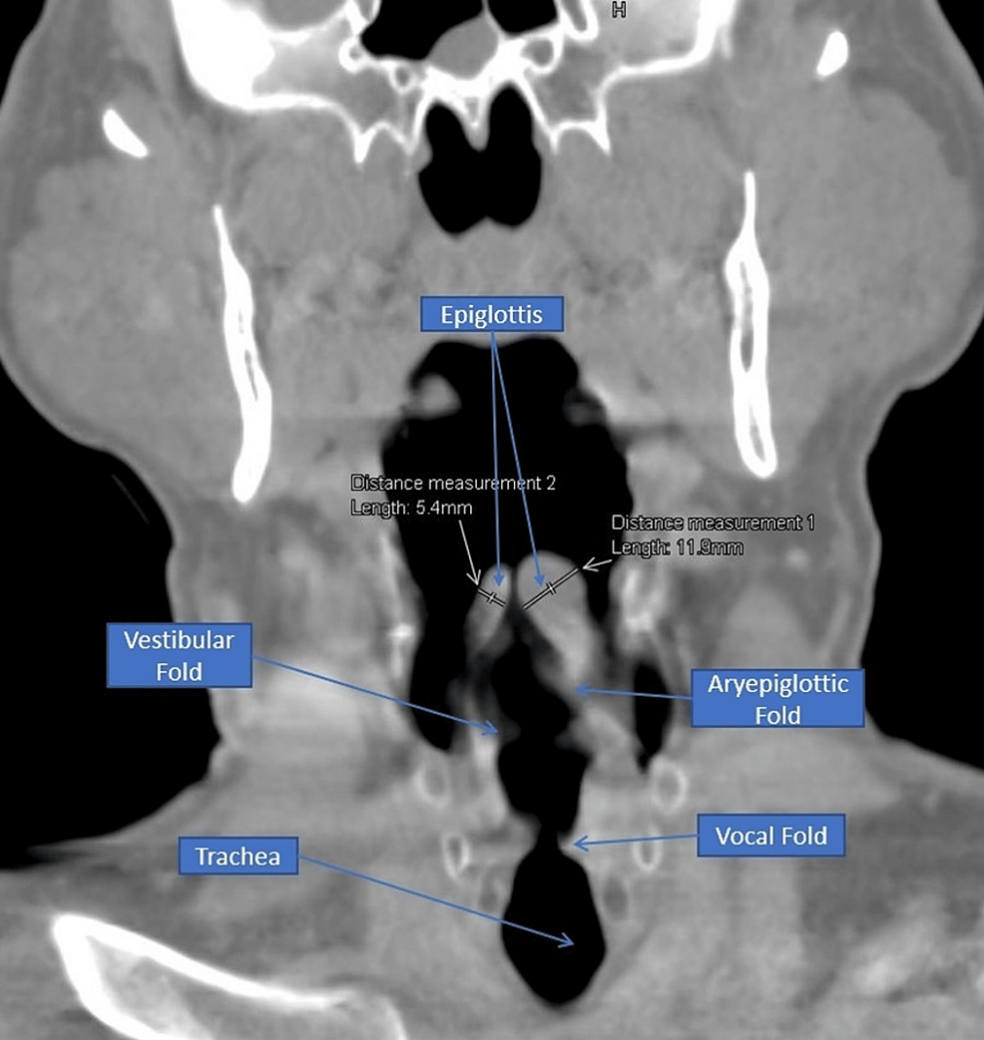
Coronal section of the CT scan neck with contrast CT: Computed Tomography

Because the patient was still complaining of neck and throat pain, the decision was made to perform a CT scan of the neck with contrast to identify the underlying pathology. Once the patient was stabilized, a trial was done to place him in a supine position while on NIPPV, which he tolerated successfully. The CT scan (Figure 2) showed a thickening of the epiglottis and aryepiglottic folds, causing severe airway narrowing.

**Figure 3:**
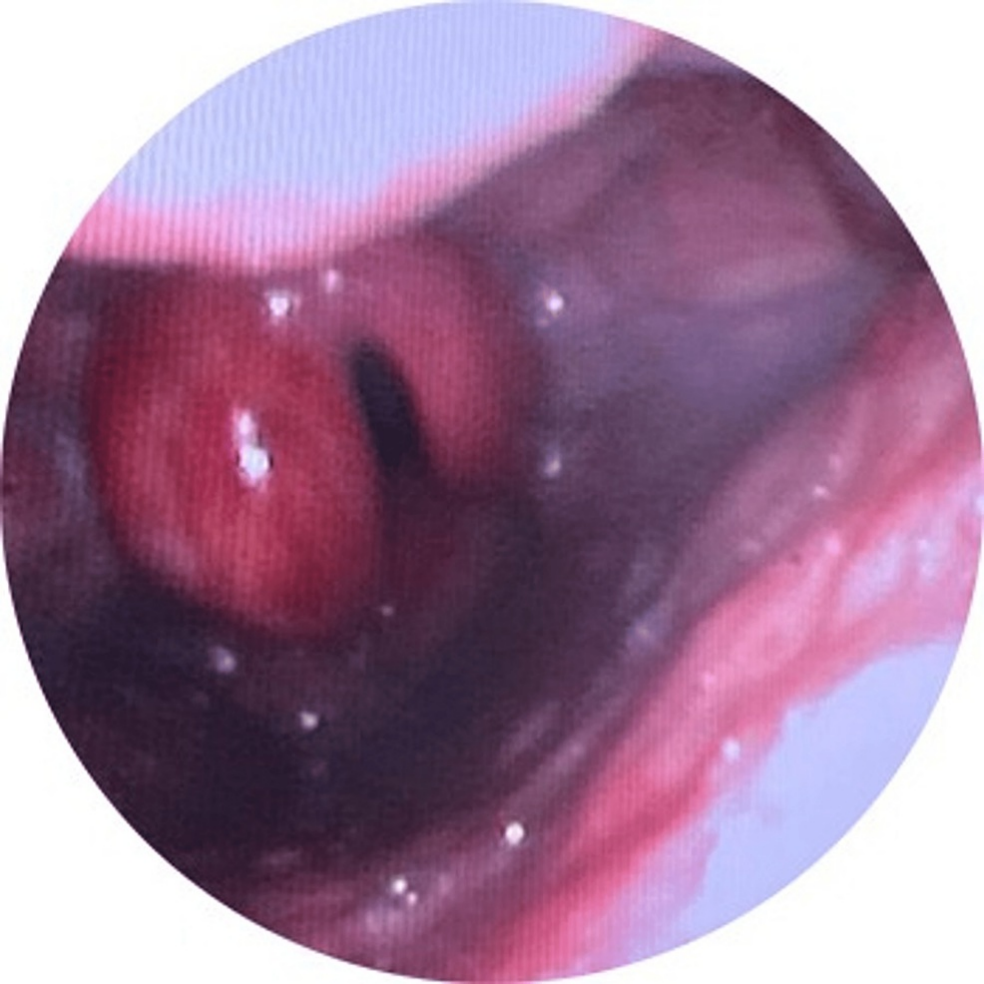
Inflamed epiglottis

The ENT physician was contacted immediately after confirmation of the CT scan finding. The patient was kept on NIPPV for four hours in the resuscitation area. When the attending staff started weaning the patient from the NIPPV, the ENT physician performed a nasopharyngeal examination using flexible nasal endoscopy. A diagnosis of acute epiglottitis was confirmed. The examination revealed severe epiglottic edema, swelling, redness of both arytenoids, and a partially obstructed airway (Figure 3). The patient was then transferred to the ICU on a nasal cannula for monitoring and discharged after five days without complications.

## Discussion

Epiglottitis is a serious condition caused by inflammation of either the epiglottis or the supraglottic structures that can leave a patient with life-threatening airway obstruction [4]. The disease was common among children in the past; however, due to the introduction of the Haemophilus influenzae vaccine in the 1990s, its prevalence has decreased significantly among that age group. Nonetheless, it can be caused by other organisms the vaccine does not protect against, such as Streptococcus pneumoniae and Haemophilus parainfluenzae, and even viruses such as Herpes simplex. Beyond infective causes, the condition can also be caused by trauma or chemical ingestions causing irritations [5]. Symptoms can include sore throat, drooling, stridor, dysphagia, and respiratory distress [4]. Due to the partial obstruction caused by the epiglottitis, our patient had a type ΙΙ respiratory failure (Table 1). In type ΙΙ (hypercapnic) respiratory failure, the PaCO_2_ is greater than 50 mmHg, and PaO_2_ may be low or normal [6].

In their study, Friedman et al. recommended a comprehensive classification for acute epiglottitis in adults based on the severity of respiratory compromise, which they classified into four stages [7]. Based on this classification, our patient was classified as stage 3, with moderate respiratory distress.

The threshold for suspecting and treating epiglottitis must be clinical and quick due to the possibility of a rapid deterioration of the patient. Physicians should remember that imaging should only be used to confirm the diagnosis [8]. Aggressiveness in treatment is required due to the swift progression of the inflammation and complete airway obstruction. Direct visualization of the epiglottis remains the gold standard for diagnosis [2]. The literature indicates that a lateral x-ray can guide the diagnosis of epiglottitis. A point not often mentioned is that obtaining this image is difficult when a patient is distressed and in a tripod position [8,9]. Most epiglottitis cases are detected clinically. Despite CT imaging not being the preferred imaging modality for diagnosis [8], it remains appropriate for diagnosis for several reasons, including practicality, accuracy in diagnosing many pathologies, and widespread availability [2]. The disadvantage of CT imaging is requiring the patient to be in the supine position. Before conducting the CT scan on a stable patient, a physician is recommended to stand by the bedside while the patient is supine as a trial to assess their tolerance [2]. Managing a patient’s airway is a top priority in acute epiglottitis. It is necessary to alert the airway management team of the possibility of a difficult airway case.

Regarding acute epiglottitis in adults, most authors advocate a more conservative management approach than the pediatric age group [10]. The need for intubation in adults can usually be determined by indirect laryngoscopy or trans-nasal fiberoptic examination of the supraglottic area [8]. Intubation is generally accomplished by “awake” fiberoptic intubation in the operating room, which should be equipped to immediately perform tracheostomy or cricothyrotomy if needed. In cases of airway obstruction in the emergency department, physicians should be prepared for difficult intubation due to the swollen, distorted anatomy [8]. In the case of intubation failure, cricothyrotomy is the only way to achieve ventilation [8]. The most experienced physician should attempt the first intubation due to the high likelihood of difficulties. Proper pre-oxygenation will give the performing physician more apnea time, which is necessary considering the anticipated difficult airway. NIPPV can be used to achieve pre-oxygenation in the event of failure with a non-rebreather mask or bag valve mask [11,12]. In our patient, we opted for NIPPV as the pre-oxygenation method because the patient could not maintain oxygen saturation above 90% on a 15 L non-rebreather mask. The patient did not initially tolerate lying in a supine position; thus, a bag valve mask was not attempted due to the inability to maintain a seal in the sitting position.

The conservative approach includes humidification and hydration, which reduce the risk of sudden airway obstruction [2]. Nebulized epinephrine and intravenous steroids are often used to reduce the inflammation and mucosal edema of the epiglottis and the surrounding structures [10].

A systematic review of randomized control trials provides level “1a” evidence for NIPPV as a treatment of type ΙΙ respiratory failure due to lower respiratory tract diseases [10]; in our case, the failure was due to partial upper airway obstruction. One case report describes the treatment of acute epiglottitis in adults using non-invasive ventilation and avoiding endotracheal intubation [10]. In such cases, the obstruction is usually for inspiration and could be bypassed with forceful ventilation [13]. Given the rarity of acute epiglottitis in adults, our case appears to be the second reported one successfully treated with NIPPV. Although NIPPV has many benefits in treating type 1 and 2 respiratory failures, clinicians should be aware that these measures may fail, especially when treating impending upper airway obstruction, resulting in the need for tracheal intubation or even cricothyrotomy.

## Conclusions

If tolerated by the patient, NIPPV can be used as a bridging therapy for pre-oxygenating an impending airway obstruction. It can be utilized with a T-piece instrument to deliver nebulized epinephrine to the inflamed structures, which may help improve the patient’s breathing efforts and decrease edema. Although intubation was avoided in our case, more data will be required prior to recommending NIPPV as a conventional method in adult epiglottitis management.
